# Global burden of ischemic heart disease associated with high red and processed meat consumption: an analysis of 204 countries and territories between 1990 and 2019

**DOI:** 10.1186/s12889-023-16954-4

**Published:** 2023-11-17

**Authors:** Dongqing Yan, Kaishan Liu, Fajun Li, Donglei Shi, Li Wei, Junhang Zhang, Xin Su, Zhaojun Wang

**Affiliations:** 1 Department of Thoracic Surgery, The Seventh Affiliated Hospital, Sun Yat-sen University, Shenzhen, China; 2 Yuhu community healthcare center, Jieyang People’s Hospital (Jieyang Affiliated Hospital, Sun Yat-sen University), Jieyang, China; 3 Department of Critical Care Medicine, The First People’s Hospital of Kunshan, Kunshan, China; 4 Department of Respiratory, Hainan Hospital of PLA General Hospital, Sanya, China

**Keywords:** Global disease burden, Red meat, Deaths, Disability-adjusted life years

## Abstract

**Background:**

Multiple studies have indicated an association between red and processed meat consumption and the incidence of ischemic heart disease (IHD). In this study, we aimed to assess the burden of IHD caused by a diet high in red and processed meat in 204 countries and territories between 1990 and 2019, using data from the Global Burden of Disease (GBD) 2019.

**Methods:**

We extracted data from the GBD 2019, which included the number of deaths, age-standardized mortality rates (ASMR), disability-adjusted life years (DALYs), and age-standardized DALYs rates (ASDR) attributed to IHD caused by a diet high in red and processed meat. We then calculated the burden of IHD attributable to a high intake of red and processed meat in each country and territory, stratified by age, sex, and socio-demographic index (SDI).

**Results:**

Globally, a high intake of red meat was responsible for 351,200 (95% uncertainty interval (UI): 559,000–642,700) deaths from IHD in 2019, while a high intake of processed meat was associated with 171,700 (95% UI: 30,100–320,000) deaths from IHD. Between 1990 and 2019, while the corresponding age-standardized rates declined, the numbers of deaths and DALYs increased. China had the highest number of deaths [98,386.9 (95% UI: 14,999.3–189,812.7)] caused by a high intake of red meat, while United States of America [33,129.6 (95% UI: 7,150–59,593.8)] was associated with the highest number of deaths caused by high intake of processed meat for IHD in 2019. Males experienced a greater burden of IHD caused by a high intake of red and processed meat than females. The ASMR and ASDR of IHD attributed to a high intake of red meat decreased in countries with high SDI, high-middle SDI and low SDI, while the ASMR and ASDR of IHD attributed to a high intake of processed meat decreased only in countries with high SDI and high-middle SDI.

**Conclusion:**

Although there is a decline in the ASMR and ASDR of IHD caused by a high intake of red and processed meat, there is also an increase in deaths and DALYs number globally. Additionally, there is a heterogeneous burden of IHD related to a high intake of red and processed meat across regions and countries, with males experiencing a greater burden than females. Implementing targeted policies and interventions is required to reduce the burden of IHD caused by a high intake of red and processed meat.

**Supplementary Information:**

The online version contains supplementary material available at 10.1186/s12889-023-16954-4.

## Background

Ischemic heart disease (IHD) is a leading cause of morbidity and mortality worldwide, constituting the most burdensome cardiovascular disease (CVD) [1]. It is a complex condition characterized by reduced blood flow to the heart muscles, primarily caused by coronary artery disease and myocardial infarction. The disease spectrum encompasses chronic stable angina, acute myocardial infarction, heart failure due to IHD, and chronic IHD [2]. The burden of IHD on sustainable development in the 21st century is significant [3, 4], and it remains a major public health challenge in both industrialized and developing countries [5]. According to the World Health Organization (WHO), IHD was responsible for over 8 million deaths in 2019, representing approximately 16% of all global deaths [6]. The Global Burden of Disease (GBD) 2019 study estimated that approximately 197 million IHD cases might have occurred globally in the same year [2]. Over the past five years, age-standardized IHD death rates have increased in some countries, including certain wealthy nations such as the United States [2]. Moreover, population aging and the escalating prevalence of obesity, diabetes and metabolic syndrome indicate that the incidence of IHD is expected to continue to rise [4].

The importance of lifestyle, particularly dietary habits, in the development of CVD and its associated risk factors has been well-documented [7]. Recent studies have pointed towards the consumption of red and processed meat as a significant contributor to the development of cardiometabolic disease [8]. However, there exists a considerable research gap in understanding the degree and mechanism through which red and processed meat consumption impacts on individual health, particularly in terms of IHD. In a significant study conducted among 25,153 California Seventh Day Adventists, regular consumption of meat was found to be associated with a 70% higher risk of fatal IHD in men and a 37% higher risk in women [9]. These alarming statistics highlight the need for further investigation into the effects of meat consumption on heart health. Furthermore, The International Agency for Research on Cancer has reclassified processed meat as a Group 1 (carcinogenic) and red meat as a Group 2A (probably carcinogenic) cancer risk [4]. These classifications provide additional impetus for this research, given the potential public health implications of meat consumption. In light of these findings, the 2015-2020 Dietary Guidelines for Americans recommend minimizing the consumption of red and processed meat [10, 11]. However, despite these guidelines, meat consumption remains high, underscoring the importance of our research in providing further evidence on the health consequences of red and processed meat. Lastly, despite a growing number of reviews suggesting that red and processed meat consumption is positively associated with both IHD and all-cause mortality [8, 12], there is a paucity of research exploring these associations in depth. Our study intends to bridge this research gap by providing a comprehensive analysis of the effects of red and processed meat consumption on IHD and all-cause mortality.

To our knowledge, there is limited global epidemiological literature on the association between a diet high in red and processed meat and IHD. In this study, we conducted a comprehensive analysis of the GBD database, published by the Institute for Health Metrics and Evaluation, to assess the global burden of IHD attributed to the high consumption of red and processed meat from 1990 to 2019. Our study aims to provide a valuable reference for policymakers and health authorities to develop effective strategies and policies for preventing and treating IHD associated with high red and processed meat intake.

## Materials and methods

### Data sources

The number of deaths, age-standardized mortality rates (ASMR), disability-adjusted life years (DALYs), and age-standardized DALY rates (ASDR) attributed to a high intake of red and processed meat and related to IHD between 1990 and 2019 were obtained from the GBD 2019 study (https://vizhub.healthdata.org/gbd-results/). Information about sex and age was also retrieved to estimate the burden of IHD. Eating habits are related to social development status. The Socio-demographic Index (SDI), created by GBD researchers and used to help generate these estimations, is a composite measure of development status highly connected with health outcomes. We downloaded the data of SDI for 21 regions in GBD 2019 (https://ghdx.healthdata.org/record/ihme-data/gbd-2019-socio-demographic-index-sdi-1950-2019).

### Definitions

The consumption of red meat, including beef, lamb, pork and goat (excluding fish, eggs, poultry, and other processed meats), was considered high if it exceeded a certain amount in grams per day. Similarly, the consumption of processed meat, which refers to meat preserved by curing, smoking, salting or adding chemical preservatives, was considered high if it exceeded a certain amount in grams per day. The 2019 GBD study set the theoretical minimum-risk exposure levels (TMREL) for harmful dietary risks to zero. But for red meat, the TMREL was set at 18-27 g/day, and for processed meat, it was set at 0-4 g/day in the 2017 GBD study. The various categories of IHD are classified according to the International Classification of Diseases (ICD) 9 codes (410-414.9, V17.3) and ICD-10 codes (I20-I21.6, I21.9-I25.9, Z82.4-Z82.49) in the 2019 GBD study. More detailed information on the 2019 GBD diagnostic and confirmation procedures can be found on the following website: https://doi.org/10.1016/j.jacc.2020.11.010. Countries and territories are categorized into five groups based on the socio-demographic index (SDI), which ranges from 0 to 1 and summarizes a country's degree of development based on its geometric average of total fertility, per capita income, and mean years of education. The five SDI categories are high (> 0.81), high-middle (0.70-0.81), middle (0.61-0.69), low-middle (0.46-0.60), and low SDI (< 0.46), and were used to divide these countries into 21 geographically GBD regions.

### Estimation methods

The GBD 2019 study utilized the comparative risk assessment framework that has been in use since 2002 to estimate the attributable burden of diseases [1, 13]. The dietary data used in the 2019 GBD study were drawn from various sources, including nationally and sub-nationally representative nutrition surveys, household budget surveys, national sales reports from Euromonitor, and data on food availability from the United Nations Food and Agriculture Organization's Supply and Utilization Accounts. The study extensively searched for published studies, household surveys, censuses, administrative data, ground monitor data, and remote sensing data relevant to each specific risk factor to obtain reliable estimates of risk exposure.

The primary data source for assessing dietary risks was 24-hour dietary recall surveys, where individuals reported or converted their food and nutrient intake into grams per person per day. Ensemble distributions were estimated for each risk based on individual-level data. Bayesian statistical models, such as spatiotemporal Gaussian process regression and DisMod-MR 2.1, specifically developed for GBD analyses over the past 12 years, were used to model exposure data. In addition, new sources from the IHME GHDx annual known survey series updates and new dietary recall sources from PubMed literature search were incorporated into the 2019 GBD study. Information on processed meat was retrieved from 41 total relative risk sources and data from 11 countries, while information for red meat was retrieved from 92 total relative risk sources and data from 20 countries [2]. All estimates were performed with a 95% uncertainty interval (UI), 95% UI was computed through the generation of 1000 simulations, utilizing the variance-covariance matrix and obtaining a random sample of the dispersion parameter from a gamma distribution. The outcomes were then summarized as the mean value of all simulations, accompanied by a corresponding 95% uncertainty interval, which consisted of the 2.5th and 97.5th percentiles of all simulations [13].

### Study design and statistical analysis

The main indicators employed to evaluate the burden of IHD attributed to high consumption of red and processed meat were deaths, DALYs, ASMR and ASDR, and the estimated annual percentage change (EAPC) between 1990 and 2019. Maps were generated to show the geographic variation of IHD burden attributed to high red and processed meat consumption based on deaths, ASMR and ASDR in 2019. The relationship between the IHD burden and SDI was evaluated based on location and year. EAPC, a concise and widely used measure of age-standardized rate trends over a specified time period, was employed [14]. The regression equation: y = α + βx + ε, was used to calculate the EAPC value, where y represents the natural logarithm of the rate, and x corresponds to the calendar year. The EAPC value was obtained as 100 × (exp (β) - 1), with a 95% confidence interval (CI) estimated using the regression model [15]. If both the EAPC value and the lower limit of its 95% CI were positive, then the rate was considered to be increasing, while if both the EAPC value and the upper limit of its 95% CI were negative, then the rate was considered to be decreasing. The data were analyzed and visualized using the R statistical software (version 4.1.2).

## Results

### Global burden of IHD attributed to diet high in red and processed meat

**Fig. 1:**
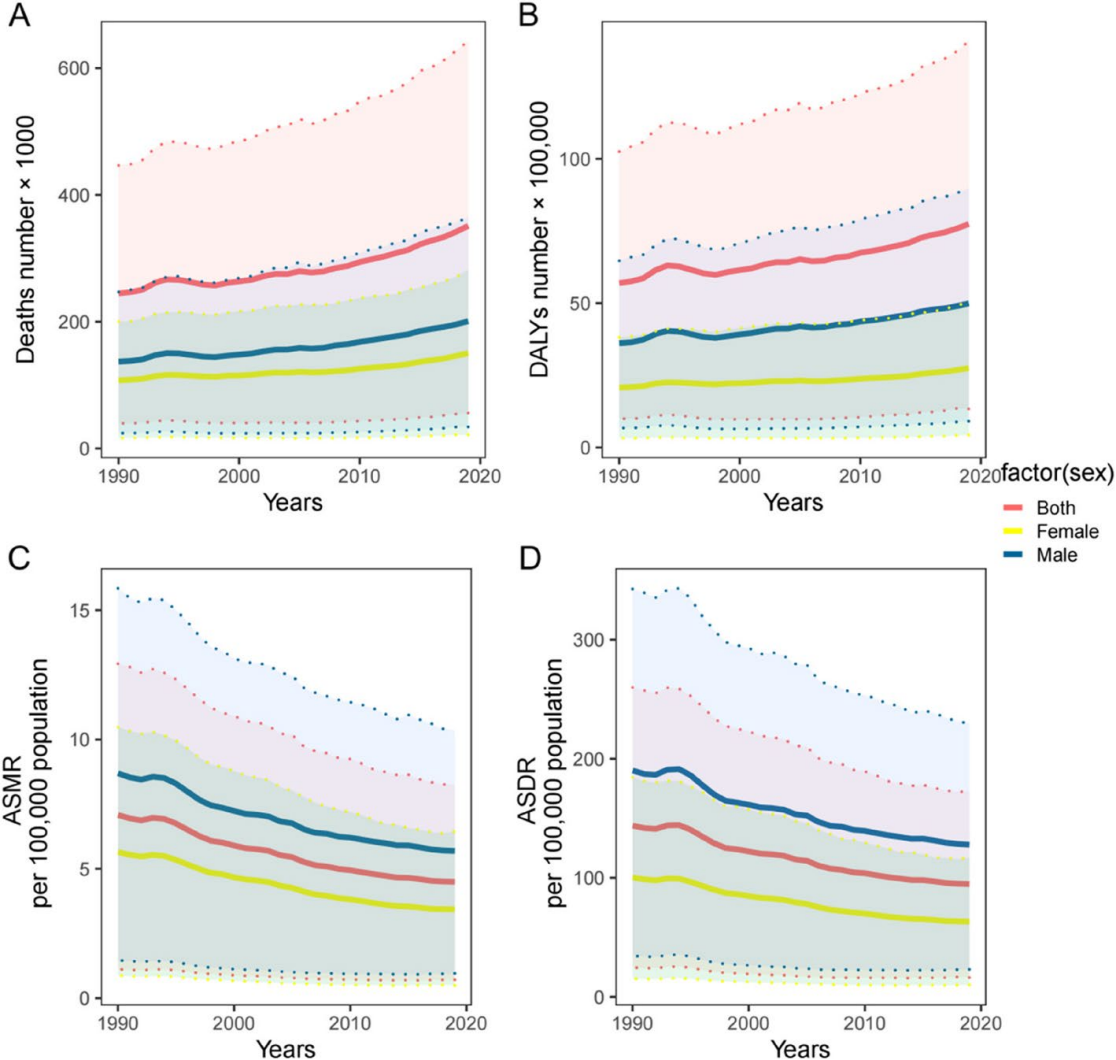
The deaths number (**A**), DALYs number (**B**), ASMR per 100000 population (**C**), ASDR per 100000 population (**D**) of ischemic heart disease attributed to diet high in red meat by sex, 1990–2019. ASMR = age-standardised mortality rate; ASDR = age-standardised DALYs rate; DALYs = disability-adjusted life years

**Fig. 2:**
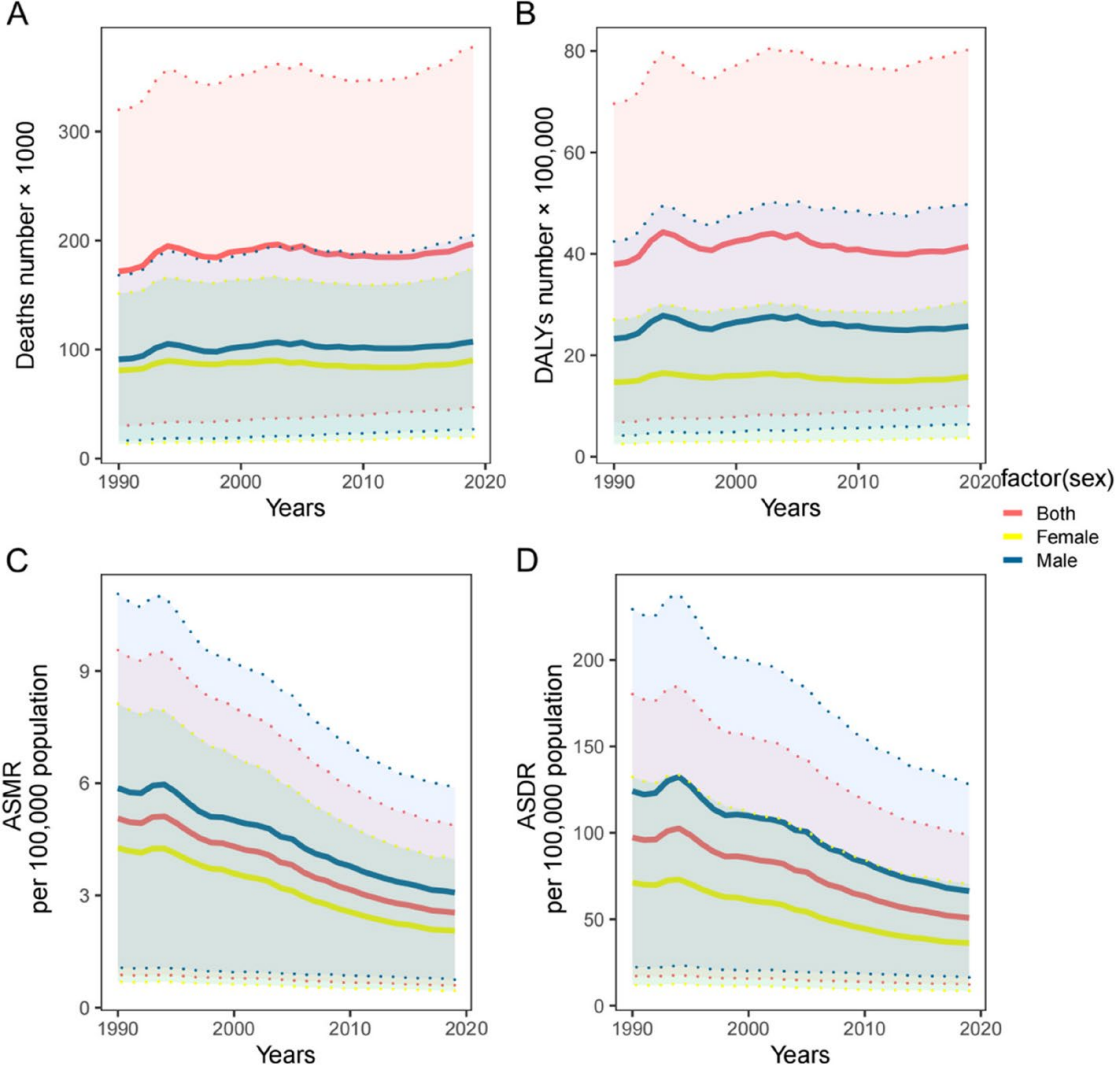
The deaths number (**A**), DALYs number (**B**), ASMR per 100,000 population (**C**), ASDR per 100,000 population (**D**) of ischemic heart disease attributed to Diet high in processed meat by sex, 1990–2019. ASMR = age-standardised mortality rate; ASDR = age-standardised DALYs rate; DALYs = disability-adjusted life years

Figures 1 and 2 show the global trends in terms of deaths, DALYs, ASMR and ASDR for IHD attributed to high red and processed meat consumption between 1990 and 2019, based on which a gradual increase in death and DALY numbers for both males and females can be observed, whereby the numbers for males were higher than females (Fig. 1A and B). Although the trend fluctuated, the death and DALY numbers for IHD attributed to high processed meat consumption remained stable (Fig. 2A and B). Both the ASMR and ASDR for IHD attributed to high red and processed meat consumption showed a downward trend, with males having significantly higher rates than both sexes and females (Figs. 1C, D and 2C, D).

In 1990, 244,400 (95% UI: 39,700–446,500) deaths were attributed to IHD caused by diet high in red meat, with an ASMR of 7.1 (95% UI: 1.1–12.9) per 100,000 population, DALY of 5,698,600 (95% UI: 996,900–10,253,700) and ASDR of 143.9 (95% UI: 24.6–259.9) per 100,000 population. In 2019, 351,200 (95% UI: 55,900–642,700) deaths were attributed to IHD caused by diet high in red meat, with an ASMR of 4.5 (95% UI: 0.7–8.3) per 100,000 population, DALY of 7,742,300 (95% UI: 134,000–14,077,700) and ASDR of 94.6 (95% UI: 16.3–172.3) per 100,000 population. From 1990 to 2019, we observed a decrease in EAPC of ASMR [-1.72 (95% CI: -1.8 to -1.63)] and ASDR [-1.62 (95% CI: -1.71 to -1.52)] (Table 1).

**Table 1:** Deaths number, ASMR, DALYs number, and ASMR of ischemic heart disease attributed to diet high in red meat in 1990 and 2019, and and its temporal trends from 1990 to 2019

location	Deaths					DALYs				
	1990 Deaths number No. ×10 3 (95% UI)	1990 ASMR per 100,000 No. (95% UI)	2019 Deaths number No. ×10 3 (95% UI)	2019 ASMR per 100,000 No. (95% UI)	1990-2019 EAPC No. (95% CI)	1990 DALYs number No. ×10 3 (95% UI)	1990 ASDR per 100,000 No. (95% UI)	2019 DALYs number No. ×10 3 (95% UI)	2019 ASDR per 100,000 No. (95% UI)	1990-2019 EAPC No. (95% CI)
Global	244.4 [39.7-446.5]	7.1 [1.1-12.9]	351.2 [55.9-642.7]	4.5 [0.7-8.3]	-1.72 (-1.8 to -1.63)	5698.6 [996.9-10253.7]	143.9 [24.6-259.9]	7742.3 [1340-14077.7]	94.6 [16.3-172.3]	-1.62 (-1.71 to -1.52)
High SDI	94.6 [18.3-170.3]	9.2 [1.8-16.5]	76.4 [13.6-139.8]	3.8 [0.7-6.8]	-3.36 (-3.56 to -3.16)	1956.4 [431.7-3434.5]	193.6 [43.2-338.5]	1390.8 [289.7-2488.4]	81.3 [18-143]	-3.21 (-3.42 to -3)
High-middle SDI	95.4 [15.4-175.7]	10.2 [1.6-18.9]	124.6 [20.6-230.5]	6.3 [1-11.7]	-2.02 (-2.18 to -1.86)	2219.7 [393.9-4053.1]	210 [36.3-382.2]	2578.4 [477.3-4672.5]	129.7 [24.4-235.2]	-2.17 (-2.38 to -1.95)
Middle SDI	34.6 [3.2-67.6]	4 [0.4-7.8]	102.3 [13.9-193.4]	4.6 [0.6-8.8]	0.85 (0.72 to 0.97)	954.9 [81.2-1869.9]	87.4 [7.8-170.9]	2469.1 [361.1-4646.6]	98.3 [14.1-185.7]	0.69 (0.59 to 0.79)
Low-middle SDI	13.7 [2.4-25.1]	2.7 [0.5-5]	35.4 [6.2-64.8]	2.8 [0.5-5.2]	0.29 (0.24 to 0.33)	388.8 [66.9-710.7]	60.4 [10.3-110.7]	938.7 [168.1-1721.2]	65.3 [11.6-119.6]	0.38 (0.33 to 0.42)
Low SDI	6 [0.7-11.8]	2.8 [0.3-5.5]	12.3 [1.5-24.2]	2.5 [0.3-4.9]	-0.33 (-0.34 to -0.31)	176.1 [17.3-346.8]	67.2 [7.1-131.4]	361.3 [37.6-715.2]	61 [6.9-119.8]	-0.34 (-0.36 to -0.33)
Andean Latin America	0.6 [0-1.1]	3 [0.3-5.8]	1.1 [0.1-2.3]	2.1 [0.2-4.2]	-1.12 (-1.34 to -0.9)	14.2 [0.9-27.8]	64.4 [4.6-125.9]	25.9 [1.9-52.6]	44.9 [3.3-91.4]	-1.17 (-1.39 to -0.95)
Australasia	3.8 [1.3-6.1]	16.9 [5.9-27]	2.8 [1-4.6]	5.2 [1.9-8.5]	-4.35 (-4.61 to -4.1)	79.7 [30.3-124.5]	346.6 [132.6-541.3]	47.1 [17.7-74.6]	101.4 [40.1-159.4]	-4.46 (-4.73 to -4.2)
Caribbean	1.3 [0.1-2.6]	5.4 [0.4-10.7]	1.9 [0.1-4]	3.7 [0.3-7.6]	-1.22 (-1.48 to -0.95)	30.9 [2-60.7]	117.3 [7.9-230.6]	43.7 [2.9-89.1]	84.6 [5.5-172.7]	-1.03 (-1.28 to -0.77)
Central Asia	6.3 [1-11.7]	14.7 [2.2-27.3]	10.1 [1.5-19.3]	16.3 [2.2-31.3]	0.1 (-0.16 to 0.35)	155.5 [27.3-282.1]	325.5 [55.9-592.1]	253.6 [41.6-476.3]	336 [53.1-636.2]	-0.24 (-0.5 to 0.02)
Central Europe	18.9 [2.3-36.1]	14.2 [1.7-27.1]	16.9 [2.3-32.4]	7.9 [1.1-15.1]	-2.29 (-2.4 to -2.18)	423.9 [57.2-792.9]	297.7 [40.4-556.6]	308.8 [51.3-574.6]	154.7 [26.4-284.7]	-2.52 (-2.66 to -2.39)
Central Latin America	3.6 [0.3-7.1]	4.8 [0.4-9.4]	8.6 [0.8-17.1]	3.7 [0.3-7.4]	-0.87 (-1.02 to -0.71)	90.9 [6.6-179.9]	101.9 [7.6-201.8]	194.2 [19.1-384.4]	80.5 [7.9-159.6]	-0.86 (-1.01 to -0.71)
Central Sub-Saharan Africa	0.5 [0.1-0.9]	2.4 [0.3-4.7]	0.9 [0.1-1.9]	1.9 [0.3-3.9]	-0.64 (-0.76 to -0.52)	13.7 [1.5-27.4]	54.7 [6.7-108.3]	26.1 [2.9-54.9]	43.7 [5.5-91.1]	-0.73 (-0.83 to -0.62)
East Asia	25.9 [2-52.8]	3.7 [0.3-7.4]	100.2 [15.3-192.7]	5.6 [0.8-10.8]	2.16 (1.9 to 2.43)	728.3 [54.5-1472.9]	80.2 [6.2-162.4]	2274.3 [425.9-4190.8]	114.4 [20.7-211.8]	1.81 (1.59 to 2.04)
Eastern Europe	43.8 [7.4-80.5]	17.3 [2.8-31.9]	42.7 [4.7-84.3]	12.6 [1.4-24.7]	-1.92 (-2.35 to -1.49)	989.9 [181.7-1779.3]	367.1 [67.4-661.1]	843.1 [89.4-1664]	256.6 [27.4-507.7]	-2.26 (-2.8 to -1.72)
Eastern Sub-Saharan Africa	1.3 [0.1-2.6]	2 [0.2-4]	2.9 [0.2-5.9]	2 [0.2-4]	-0.1 (-0.13 to -0.07)	38.8 [3.4-76.8]	47.1 [4.5-93.2]	80.9 [5.9-169.3]	44.2 [3.7-91.8]	-0.25 (-0.28 to -0.22)
High-income Asia Pacific	4.7 [0.4-9.4]	2.6 [0.2-5.2]	5.3 [0.5-10.7]	1.1 [0.1-2.1]	-3.2 (-3.39 to -3.02)	102.8 [6.5-204]	52.2 [3.4-103.8]	87.5 [8.4-170.1]	23.1 [2.4-44.1]	-2.79 (-2.95 to -2.64)
High-income North America	38.2 [7.7-69]	10.8 [2.3-19.4]	35.6 [7.5-64.4]	5.5 [1.2-9.9]	-2.73 (-2.92 to -2.54)	803.1 [183.1-1398.7]	240.5 [55.9-417.7]	688 [162.3-1207.1]	120.1 [29.3-208.1]	-2.69 (-2.87 to -2.51)
North Africa and Middle East	13.2 [1-26.1]	8.3 [0.7-16.3]	23 [1.8-46.2]	5.7 [0.5-11.4]	-1.36 (-1.49 to -1.24)	372.5 [24.1-742.8]	196.8 [14-390.1]	608.5 [39.9-1241.6]	128.3 [9.2-259.3]	-1.61 (-1.74 to -1.49)
Oceania	0.2 [0-0.4]	6.9 [0.5-14.5]	0.5 [0-1]	6.9 [0.5-14.3]	-0.03 (-0.05 to -0.01)	6.8 [0.4-14.4]	186.4 [11.7-392.5]	15.8 [0.9-33.9]	181 [11-382.9]	-0.11 (-0.14 to -0.08)
South Asia	8.9 [2.1-15.9]	1.8 [0.4-3.2]	22.8 [4.7-42]	1.7 [0.4-3.2]	-0.22 (-0.28 to -0.15)	265.1 [59.8-475.4]	42 [9.7-75.4]	635.6 [119.2-1183.6]	42.1 [8.2-78.2]	-0.03 (-0.1 to 0.05)
Southeast Asia	4.9 [0.6-9.4]	2.1 [0.3-4]	15.1 [1.4-29.5]	2.7 [0.3-5.2]	0.89 (0.77 to 1)	140 [13.9-272.1]	49.4 [5.4-95.2]	412.1 [34.3-809.5]	63.3 [5.6-124.1]	0.98 (0.86 to 1.1)
Southern Latin America	5.9 [2.1-9.6]	13.8 [4.8-22.4]	5.3 [1.8-8.5]	6.3 [2.1-10.1]	-2.67 (-2.93 to -2.41)	133.9 [50.8-210.7]	293.1 [110.9-464]	107.8 [41.3-170.6]	133.1 [51.9-210.2]	-2.7 (-2.94 to -2.47)
Southern Sub-Saharan Africa	0.9 [0.1-1.8]	3.3 [0.2-6.8]	1.8 [0.1-3.5]	3.5 [0.3-7]	0.3 (-0.08 to 0.68)	24.6 [1.6-50]	80.7 [5.4-164.2]	45.1 [3.5-89.7]	76 [6-151.6]	-0.05 (-0.45 to 0.36)
Tropical Latin America	6.2 [0.7-12]	7.5 [0.8-14.5]	14.3 [4.2-23.9]	5.9 [1.7-10]	-0.52 (-0.74 to -0.3)	171.7 [19.2-329.9]	173.2 [19-334.4]	372.8 [124.7-611.5]	149.5 [49.4-245.6]	-0.25 (-0.53 to 0.05)
Western Europe	53.3 [10.2-95.8]	9.4 [1.8-16.8]	35.6 [5.8-66]	3.5 [0.6-6.4]	-3.75 (-3.95 to -3.56)	1064.2 [230.1-1865.9]	196 [44.3-341.1]	568.3 [108.9-1023.3]	68 [14.5-119.9]	-3.97 (-4.17 to -3.76)
Western Sub-Saharan Africa	1.9 [0.2-3.8]	2.4 [0.3-4.9]	4 [0.4-8.1]	2.4 [0.2-5]	0.11 (0.06 to 0.16)	48 [3.9-98.5]	52.5 [4.7-106.9]	103.1 [7.6-214.1]	51.2 [4.4-105]	-0.02 (-0.08 to 0.05)

*ASMR* Age-standardized death rates,*DALYs* Disability-adjusted life years, *ASDR* Age-standardized DALYs rates, *EAPC* Estimated annual percentage change, *UI* Uncertainty intervals, *CI* Confidence interval

In 1990 and 2019, 171,700 (95% UI: 30,100–320,000) and 197,200 (95% UI: 47,000–377,600) deaths were attributed to IHD caused by a diet high in processed meat, respectively, with a corresponding DALY of 3,792,600 (95% UI: 676,400–6,961,700) and ASDR of 97.3 (95% UI: 17.2–180.3) per 100,000 population in 1990, and a DALY of 4,141,900 (95% UI: 998,600–8,024,100) and ASDR of 50.8 (95% UI: 12.2–98.3) per 100,000 population in 2019, and the EAPC of ASMR [-2.67 (95% CI: -2.8 to -2.53)] and ASDR [-2.57 (95% CI: -2.74 to -2.4)] of IHD attributed to high processed meat diet from 1990 to 2019 (Table 2).

**Table 2:** Death number, DALYs number, ASMR and ASDR of ischemic heart disease attributed to diet high in processed meat in 1990 and 2019, and and its temporal trends from 1990 to 2019

Location	Deaths					DALYs				
	1990 Deaths number No. ×10 3 (95% UI)	1990 ASMR per 100,000 No. (95% UI)	2019 Deaths number No. ×10 3 (95% UI)	2019 ASMR per 100,000 No. (95% UI)	1990-2019 EAPC No. (95% UI)	1990 DALYs number No. ×10 3 (95% UI)	1990 ASDR per 100,000 No. (95% UI)	2019 DALYs number No. ×10 3 (95% UI)	2019 ASDR per 100,000 No. (95% UI)	1990-2019 EAPC No. (95% CI)
Global	171.7 [30.1-320]	5.1 [0.9-9.6]	197.2 [47-377.6]	2.5 [0.6-4.9]	-2.67 (-2.8 to -2.53)	3792.6 [676.4-6961.7]	97.3 [17.2-180.3]	4141.9 [998.6-8024.1]	50.8 [12.2-98.3]	-2.57 (-2.74 to -2.4)
High SDI	81.7 [9.9-152.4]	7.9 [1-14.8]	70.6 [9.2-128.9]	3.5 [0.4-6.3]	-3.13 (-3.25 to -3)	1638.9 [198.3-3011.4]	161.3 [19.4-295.8]	1268.8 [166.6-2269]	73 [9.4-129.4]	-2.96 (-3.07 to -2.86)
High-middle SDI	68.7 [10-126.8]	7.3 [1.1-13.6]	67.2 [10.7-138]	3.4 [0.5-7]	-3.12 (-3.48 to -2.76)	1559.8 [217.4-2841.4]	147.7 [20.9-270.5]	1329.6 [208-2698.9]	66.6 [10.4-135.1]	-3.39 (-3.86 to -2.92)
Middle SDI	9.8 [4.4-20.4]	1.1 [0.5-2.4]	29.6 [12.3-63.7]	1.3 [0.6-2.8]	0.71 (0.61 to 0.82)	260.7 [111-557.8]	24.3 [10.6-50.9]	721.4 [275.2-1609.7]	28.6 [11.2-63.4]	0.81 (0.71 to 0.91)
Low-middle SDI	8.2 [3.7-15.7]	1.5 [0.7-3]	22.3 [10.1-43.4]	1.7 [0.8-3.4]	0.63 (0.55 to 0.7)	238.2 [105.8-456.1]	36.1 [16-69.7]	600.4 [258.7-1190.8]	41.4 [18.1-81.6]	0.67 (0.58 to 0.75)
Low SDI	3.2 [0.8-8.1]	1.5 [0.4-3.8]	7.5 [2-18.6]	1.5 [0.4-3.8]	0.07 (0.01 to 0.13)	93.8 [22.8-237.5]	35.7 [9-90.9]	220.4 [51.3-549.7]	37 [9.3-92.4]	0.11 (0.03 to 0.19)
Andean Latin America	0.1 [0.1-0.2]	0.6 [0.3-1.1]	0.2 [0.1-0.5]	0.4 [0.2-0.8]	-1.17 (-1.43 to -0.91)	2.8 [1.3-5.9]	12.6 [6.3-25.5]	5.2 [1.6-12.8]	8.9 [3-21.7]	-1.09 (-1.35 to -0.83)
Australasia	1.5 [0.1-3.2]	6.7 [0.5-14.5]	1.3 [0.1-2.7]	2.5 [0.2-5]	-3.59 (-3.8 to -3.38)	31.5 [2.3-65.7]	137.9 [9.9-287.3]	23 [2-44.6]	50 [4.2-95.6]	-3.57 (-3.79 to -3.35)
Caribbean	0.4 [0.1-1]	1.5 [0.4-4.1]	0.6 [0.1-1.6]	1.1 [0.3-3.1]	-1.19 (-1.43 to -0.96)	8.9 [1.9-24.7]	33.7 [7.4-93]	12.9 [2.5-37.7]	25 [4.8-73]	-1 (-1.24 to -0.77)
Central Asia	3.3 [0.3-8.1]	7.6 [0.7-18.9]	5.4 [0.5-13]	8.4 [0.8-20.8]	0.03 (-0.26 to 0.33)	83.8 [6.4-194.6]	173.8 [13.7-408.7]	138.1 [10.8-332]	179.6 [15.4-429.6]	-0.32 (-0.63 to 0)
Central Europe	8.4 [0.9-20.8]	6.4 [0.7-15.8]	9.7 [0.9-21.5]	4.6 [0.4-10]	-1.22 (-1.31 to -1.13)	190 [18-463.8]	134 [12.7-325.6]	184.9 [15.9-393.8]	93.8 [7.9-197.1]	-1.23 (-1.34 to -1.12)
Central Latin America	0.9 [0.2-2.5]	1.2 [0.3-3.2]	2.4 [0.5-6.3]	1 [0.2-2.7]	-0.65 (-0.83 to -0.48)	23.8 [4.6-65.4]	26.4 [5.7-71.4]	53.7 [10.4-147.5]	22.2 [4.4-61.1]	-0.62 (-0.81 to -0.43)
Central Sub-Saharan Africa	0.3 [0-0.9]	1.4 [0.3-4.2]	0.5 [0.1-1.7]	1.2 [0.2-3.4]	-0.67 (-0.71 to -0.63)	8.1 [1.1-25.4]	32.4 [5.1-100.3]	15.5 [2.2-48.8]	26.1 [4.2-79.4]	-0.81 (-0.85 to -0.77)
East Asia	4 [1.9-8.2]	0.6 [0.3-1.2]	15.9 [4.5-41.6]	0.9 [0.3-2.3]	2.26 (1.96 to 2.56)	107.5 [46.6-231.5]	12.2 [5.6-25.4]	353.3 [79.2-974.6]	17.8 [4.2-48.8]	2.06 (1.78 to 2.33)
Eastern Europe	49.3 [5.8-90.3]	19.2 [2.3-35]	40.1 [5.3-79.7]	11.7 [1.5-23.4]	-2.36 (-2.94 to -1.78)	1120.9 [125-2093.1]	413.8 [46.4-769.2]	779.2 [95.5-1528.2]	235.9 [28.4-463.4]	-2.77 (-3.49 to -2.05)
Eastern Sub-Saharan Africa	0.7 [0.1-1.9]	1 [0.2-2.8]	1.4 [0.2-4.2]	1 [0.2-2.8]	-0.12 (-0.15 to -0.1)	19.1 [3-56.3]	23.1 [4.1-67]	39 [5.7-119.3]	21.3 [3.6-64]	-0.31 (-0.35 to -0.28)
High-income Asia Pacific	4.2 [0.5-9.2]	2.2 [0.3-4.8]	4.4 [0.6-9.6]	1 [0.1-2]	-2.75 (-2.91 to -2.58)	100 [10.6-210.1]	49.3 [5.3-104]	84.3 [9.7-173.9]	23.2 [2.7-46.1]	-2.43 (-2.64 to -2.22)
High-income North America	33.7 [4-62.3]	9.5 [1.1-17.5]	35.6 [4.7-63.3]	5.5 [0.7-9.7]	-2.28 (-2.44 to -2.12)	694.8 [82.1-1254.5]	206.8 [24.2-373.4]	687.4 [84.7-1191.8]	119.2 [14.8-207.4]	-2.2 (-2.34 to -2.06)
North Africa and Middle East	3.3 [1.1-8.2]	2.1 [0.8-5.1]	6.6 [1.8-17.7]	1.7 [0.5-4.3]	-0.89 (-0.97 to -0.81)	90.8 [25.4-235.5]	48.7 [15-123.1]	173.4 [39.7-485.6]	36.8 [9.4-100.7]	-1.04 (-1.12 to -0.96)
Oceania	0 [0-0.1]	1.2 [0.5-2.8]	0.1 [0-0.2]	1.4 [0.5-3.2]	0.42 (0.37 to 0.46)	1.2 [0.3-2.9]	32.6 [10.6-78.7]	3.1 [0.8-8]	35.5 [10.3-89.5]	0.36 (0.3 to 0.42)
South Asia	9.2 [4.9-15.7]	1.8 [1-3.2]	28.4 [16-46.8]	2.2 [1.2-3.5]	0.76 (0.66 to 0.86)	271.8 [143.4-461.1]	42.9 [22.7-74]	770.5 [416.9-1302.9]	51.5 [28.3-86.1]	0.82 (0.72 to 0.92)
Southeast Asia	1.2 [0.8-2]	0.5 [0.4-0.9]	3.8 [1.8-8.1]	0.7 [0.3-1.4]	0.94 (0.86 to 1.03)	33.1 [20.5-57.8]	12 [7.7-20.5]	101.7 [43-231.8]	15.8 [7-35.1]	1.2 (1.1 to 1.3)
Southern Latin America	1.5 [0.2-3.7]	3.5 [0.4-8.8]	1.8 [0.2-4.1]	2.2 [0.2-4.9]	-1.56 (-1.81 to -1.32)	33.2 [3.1-81.7]	73.1 [6.9-180.7]	38.4 [4-84]	47.6 [4.9-103.8]	-1.34 (-1.58 to -1.1)
Southern Sub-Saharan Africa	0.2 [0-0.6]	0.8 [0.2-2.3]	0.5 [0.1-1.3]	0.9 [0.2-2.5]	0.47 (0.1 to 0.83)	5.9 [1.1-17.4]	19.5 [3.9-56.7]	11.7 [2-34.9]	19.7 [3.6-57.8]	0.19 (-0.2 to 0.58)
Tropical Latin America	1.2 [0.3-3.4]	1.5 [0.3-4]	2.2 [0.4-6.4]	0.9 [0.2-2.7]	-1.29 (-1.5 to -1.08)	33.5 [6.1-96.5]	33.7 [6.7-96]	58 [8.2-167.4]	23.2 [3.4-67]	-0.99 (-1.19 to -0.79)
Western Europe	46.8 [5.7-87.1]	8.2 [1-15.2]	33.2 [4.2-62.1]	3.2 [0.4-6]	-3.51 (-3.64 to -3.37)	894.8 [107.2-1633.9]	162.8 [19.3-296.5]	523.3 [62.8-940.8]	61.6 [7.6-110.6]	-3.62 (-3.75 to -3.48)
Western Sub-Saharan Africa	1.4 [0.2-4.1]	1.8 [0.3-5.3]	3.2 [0.4-8.5]	1.9 [0.2-5.2]	0.18 (0.09 to 0.27)	37.1 [4.1-108]	40.1 [4.8-116.3]	85.4 [8.2-228.6]	41.2 [4.5-110.6]	0.12 (0 to 0.24)

*ASMR* Age-standardized death rates, *DALYs* Disability-adjusted life years, *ASDR* Age-standardized DALYs rates, *EAPC* estimated annual percentage change, *UI* Uncertainty intervals, *CI* Confidence interval

### Regional and national burden of IHD attributed to high red and processed meat consumption

Among the 21 regions classified by geography in the GBD study, East Asia [100,200 (95% UI: 15,300–192,700)] had the highest number of deaths due to IHD in 2019. Between 1990 and 2019, the number of deaths decreased in only six regions (Australasia, Central Europe, Eastern Europe, High-income North America, Southern Latin America, and Western Europe). Central Asia had the highest ASMR [16.3 (95% UI: 2.2–31.3)] per 100,000 population in 2019, while high-income Asia Pacific had the lowest ASMR [1.1 (95% UI: 0.1–2.1)]. Comparatively, East Asia had the highest EAPC of ASMR for IHD [2.16 (95% CI: 1.9 to 2.43)], while Australasia had the lowest [-4.35 (95% CI: -4.61 to -4.1)]. The EAPC of ASMR for IHD caused by high red meat consumption increased in five regions (Central Asia, East Asia, Southeast Asia, Southeast Asia, and Western Sub-Saharan Africa) between 1990 and 2019. East Asia also had the highest number of DALYs [2,274,300 (95% UI: 425,900–4,190,800)], while Oceania had the lowest number of DALYs [15,800 (95% UI: 900–33,900)] in 2019. Further, Central Asia had the highest ASDR [336 (95% UI: 53.1–636.2)] per 100,000 population in 2019, whereas high-income Asia Pacific had the lowest ASDR [23.1 (95% UI: 2.4–44.1)]. A decreasing trend of ASDR was detected in 19 regions between 1990 and 2019, while the EAPC of ASDR is increasing in only two regions (East Asia and Southeast Asia) (Table 1).

Eastern Europe [40,100 (95% UI: 5,300–79,700)] had the highest number of deaths, followed by high-income North America [35,600 (95% UI: 4,700–63,300)] and Western Europe [33,200 (95% UI: 4,200–62,100)] in 2019. Similarly, Eastern Europe [11.7 (95% UI: 1.5–23.4)] had the highest ASMR of IHD attributed to high processed meat consumption, while Andean Latin America [0.4 (95% UI: 0.2–0.8)] had the lowest ASMR per 100,000 population in 2019. East Asia [2.26 (95% CI: 1.96 to 2.56)] had the highest EAPC of ASMR, while Australasia [-3.59 (95% CI: -3.8 to -3.38)] had the lowest. The EAPC of ASMR had increased in seven regions and decreased in another 14 regions between 1990 and 2019. Eastern Europe [779.2 (95% UI: 95.5–1528.2)] and South Asia [770.5 (95% UI: 416.9–1302.9)] had the highest DALYs, Eastern Europe [235.9 (95% UI: 28.4–463.4)] and Central Asia [179.6 (95% UI: 15.4–429.6)] had the highest ASDR per 100,000 population. The EAPC of ASDR attributed to diet high in processed meat decreased most in Western Europe [-3.62 (95% CI: -3.75 to -3.48)] and Australasia [-3.57 (95% CI: -3.79 to -3.35)], while the most significant increase was observed in East Asia [2.06 (95% CI: 1.78 to 2.33)]. Growth was also observed in five other regions, namely Oceania, South Asia, Southeast Asia, Southern Sub-Saharan Africa and Western Sub-Saharan Africa (Table 2).

**Fig. 3:**
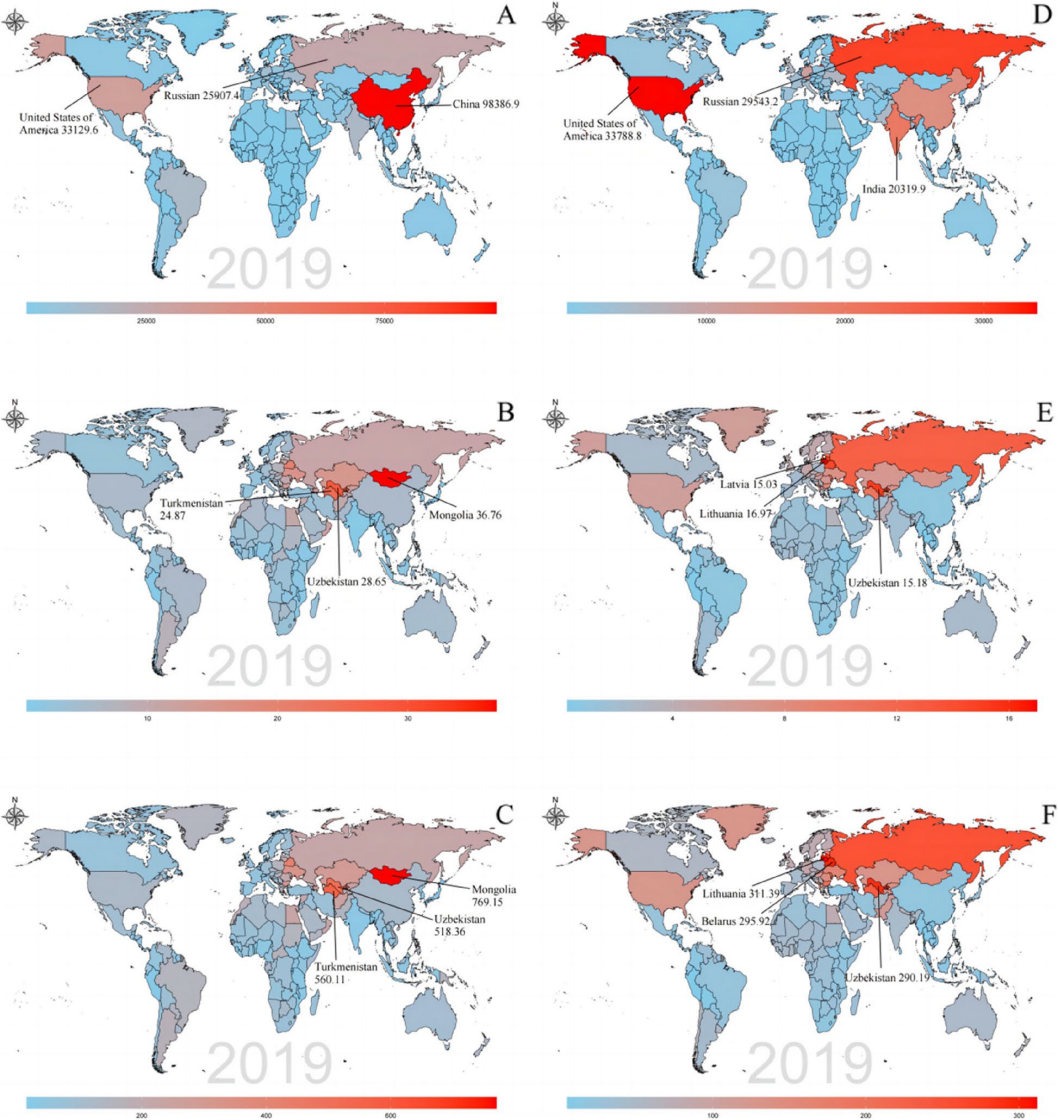
Global distribution of ischemic heart disease attributed to diet high in red meat burden in terms of deaths (**A**), ASMR (B) and ASDR (**C**) , and attributed to diet high in red meat burden in terms of deaths (**D**), ASMR (**E**) and ASDR (**F**) in 2019. ASMR = age-standardized mortality rate; ASDR = age-standardized DALYs rate; DALY= disability-adjusted life year

Among 204 countries and territories, China [98,386.9 (95% UI: 14,999.3–189,812.7)] had the highest number of IHD death attributed to high red meat consumption in 2019, followed by the United States of America [33,129.6 (95% UI: 7,150–59,593.8)] and Russia [25,907.4 (95% UI: 2,954.8–50,910.7)] (Fig. 3A and Additional file 1). In contrast, the United States of America [33,788.8 (95% UI: 4,505.2–60,185.8)] had the highest death number of IHD attributed to high processed meat diet in 2019, followed by Russia [29,543.2 (95% UI: 3,844.1–56,331)] and India [20,319.9 (95% UI: 13,520.6–29,876.6)] (Fig. 3D and Additional file 4). In four countries (Tuvalu, Nauru, Niue, and Tokelau), as well as 16 other countries (Bermuda, Grenada, Tonga, Seychelles, Dominica, Marshall Islands, Antigua and Barbuda, Northern Mariana Islands, American Samoa, Saint Kitts and Nevis, Palau, Cook Islands, Tuvalu, Nauru, Niue, and Tokelau), the number of deaths from IHD attributed to diet high in red and processed meat was less than one (Additional files 1 and 4). The highest ASMR of IHD was recorded in Mongolia, at 36.76 (95% UI: 16.38–58.05) per 100,000 population, followed by Lithuania at 16.97 (95% UI: 2.19–31.32) per 100,000 population (Fig. 3B and E). The ASMR of IHD attributed to high red and processed meat diet was less than 1 in 3 and 53 countries (Additional files 2 and 5). The highest ASDR of IHD attributed to high red meat diet in 2019 occurred in Mongolia [769.15 (95% UI: 359.47–1,196.4)], Turkmenistan [560.11 (95% UI: 150.24–981.49)] and Uzbekistan [518.36 (95% UI: 38.12–1,067.45)] (Fig. 3C and Additional file 3). The highest ASDR of IHD attributed to a diet high in processed meat was recorded in Lithuania [311.39 (39.44–592.55)], followed by Belarus [295.92 (95% UI: 25.8–589.08) ] and Uzbekistan [290.19 (95% UI: 25.24–734.05)] per 100,000 population (Fig. 3F and Additional file 6).

### Age and sex patterns

**Fig. 4:**
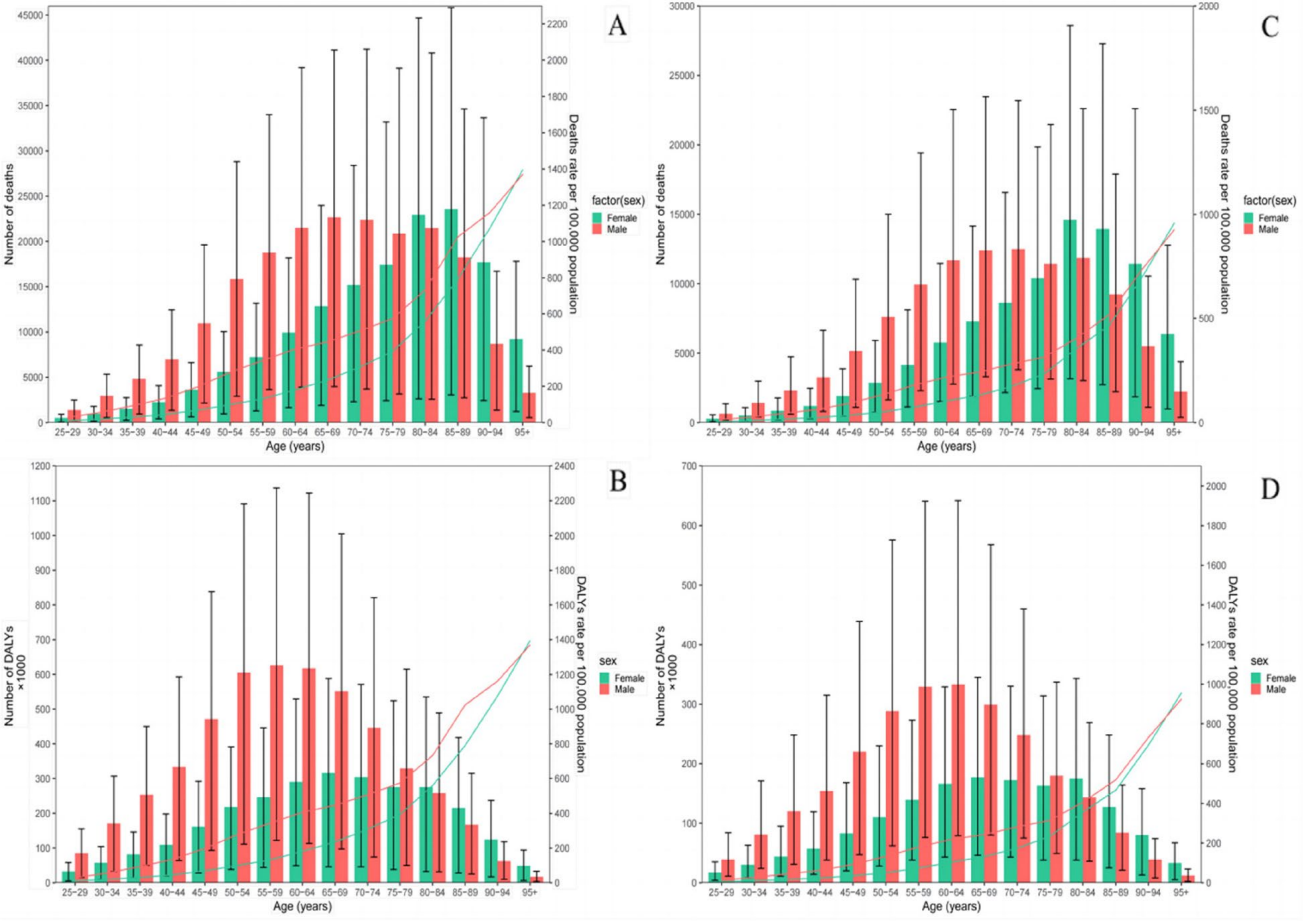
Global number of deaths and death rate (**A**) and global number of DALYs and DALY rate per 100,000 population (**B**) of ischemic heart disease attributed to diet high in red meat, and global number of deaths and death rate (**C**) and global number of DALYs and DALY rate per 100,000 population (**D**) of ischemic heart disease attributed to diet high in processed meat by age and sex in 2019. DALY, disability-adjusted life years

The number of deaths and DALYs of IHD attributed to a diet high in red and processed meat were higher in males than females before 75-79 years old, after which the number of females began to surpass males (Fig. 4). In males, the number of deaths attributed increased with age and peaked at 65-69 and 70-74 years old, followed by a declining trend except for the 80-84 age group. In females, the number of deaths increased, peaking at 85-89 and 80-84 years old, then demonstrated a decreasing trend (Fig. 4A and C). The DALYs of IHD attributed to a diet high in red and processed meat increased in males, peaking at 55-59 and 60-64 years old (Figures 4B and D), and in females, the DALYs also showed an increasing trend and peaked at 65-69 years old, before starting to decrease except for the 80-84 age group (Fig. 4B). The DALYs attributed to high processed meat diet showed a discontinuous increasing trend in females, peaking at 80-84 years old (Fig. 4D), while it increased with age and peaking at 55-59 years old in males before declining, while in females, it peaked at 65-69 years old. The age-specific rates of deaths and DALYs increased with age, with males having higher rates than females until the age of 95+ years, after which females had higher rates than males (Fig. 4).

### Association with the SDI

The number of deaths and DALYs attributed to high red meat diet decreased in the high SDI region, while it increased in the other four SDI regions. In 2019, the corresponding high-middle SDI region had the highest number of deaths [124,600 (95% UI: 20,600–230,500)], DALYs [2,578.4 (95% UI: 477.3–4,672.5)], ASMR [6.3 (95% UI: 1–11.7)] per 100,000 population, and ASDR [129.7 (95% UI: 24.4–235.2)] per 100,000 population. On the other hand, Low SDI regions had the lowest number of deaths, DALYs, ASMR and ASDR. The lowest EAPC of ASMR and ASDR of IHD attributed to diet high in red meat were in High SDI, while Middle SDI and Low-middle SDI show an increased trend (Table 1).

In 2019, the highest number of IHD deaths attributed to diet high in processed meat was observed in High SDI with 70,600 (95% UI: 9,200–128,900) deaths. However, the deaths and DALYs decreased in both High SDI and High-middle SDI regions, while they increased in the other three regions. High-middle SDI had the highest IHD DALYs attributed to diet high in processed meat in 2019, [1,329,600 (95% UI: 208,000–2,698,900)]. The lowest EAPC of ASMR [-3.13 (95% CI: -3.25 to -3)] of IHD attributed to diet high in processed meat was observed in High SDI between 1990 and 2019, while the lowest EAPC of ASDR [-3.39 (95% CI: -3.86 to -2.92)] was observed in High-middle SDI (Table 2).

**Fig. 5:**
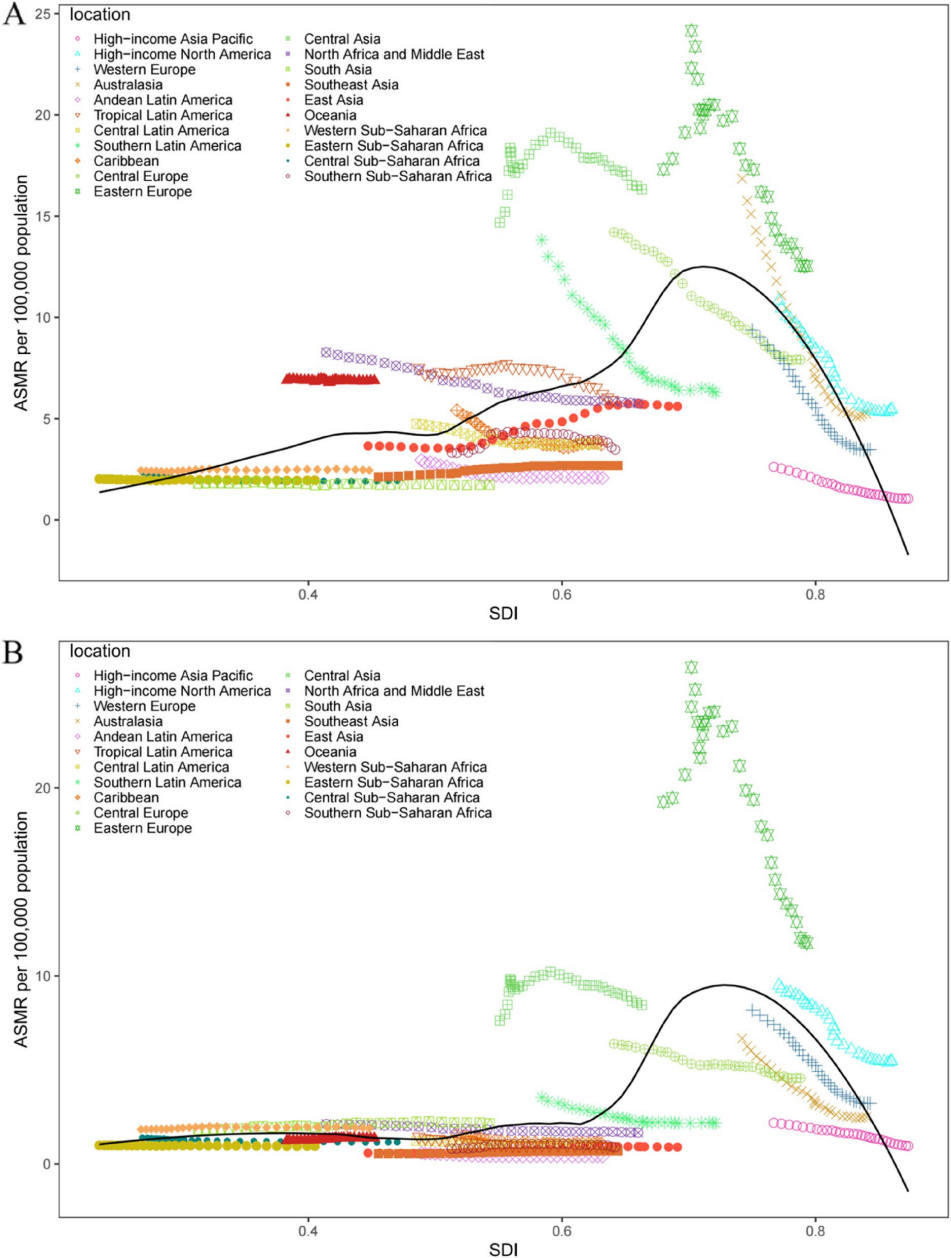
Age-standardised mortality rates (ASMR) of ischemic heart disease attributed to diet high in red meat (**A**) and diet high in processed meat (**B**) for 21 GBD regions by Socio-demographic Index, 1990-2019. Expected values based on Socio-demographic Index and disease rates in all locations are shown as the black line. DALY=disability-adjusted life-year; GBD=Global Burden of Diseases

Next, we explored the relationship between SDI and the corresponding ASMR of IHD attributed to diet high in red and processed meat in 21 GBD regions from 1990 to 2019. The results showed an n-shape association between the regional SDI and the corresponding ASMR of IHD attributed to diet high in red meat from 1990 to 2019. When SDI was less than 0.7, the ASMR of IHD attributed to diet high in red meat gradually increased with the increase in SDI, then decreased substantially. Central Asia, Oceania, Central Europe and High-income North America had higher than expected burdens of ASMR of IHD attributed to diet high in red meat from 1990 to 2019. On the contrary, the ASMR of IHD attributed to diet high in red meat was lower than expected for Andean Latin America, South Asia, Southeast Asia and Southern Sub-Saharan Africa. In the early years of the measurement period, Tropical Latin America, North and Middle East Africa, Caribbean, Central Latin America, Western Sub-Saharan Africa, Eastern Sub-Saharan Africa, Southern Latin America and Central Europe had a higher burden of ASMR than expected but then decreased during the later years (Fig. 5A).

A non-linear relationship was observed between the regional SDI and the corresponding ASMR of IHD attributed to diet high in processed meat before the SDI was less than 0.6. From 1990 to 2019, an n-shaped association was observed. Western Sub-Saharan Africa, South Asia, Central Asia, Eastern Europe and High-income North America had a higher burden than expected of ASDR of IHD attributed to diet high in processed meat, while that of Australasia, Eastern Sub-Saharan Africa, East Asia, Southern Sub-Saharan Africa, Southeast Asia, Andean Latin America and Central Latin America were lower than expected burdens. In the early years of the measurement period, North and Middle East Africa, Southern Latin America and Central Europe had a higher burden of ASDR than expected, but their burden decreased during the later years. Conversely, Western Europe and Southern Sub-Saharan Africa had a lower burden of ASDR than expected in the early years of the measurement period but then increased during the later years (Fig. 5B). Australasia, Eastern Europe, Western Europe, High-income Asia Pacific and High-income North America were the five regions where ASMR gradually decreased as SDI increased (Fig. 5A and B).

## Discussion

This present study systematically examines the latest estimates of temporal trends in the death and DALY burden of IHD attributable to diet high in red and processed meat from 1990 to 2019 at global, regional and national levels. The results showed that the ASMR and ASDR associated with IHD attributed to red and processed meat decreased between 1990 and 2019 globally, while the number of deaths and DALYs increased. However, the trends in ASMR and ASDR varied among SDI regions and were heterogeneous across regional and national burdens. Additionally, the number of deaths and DALYs differed among age groups, with age-specific rates of deaths and DALYs increasing with age, among which males had higher rates than females. Therefore, targeted and continuous efforts are necessary to reduce the death and DALY burden of IHD attributed to red and processed meat.

CVD has numerous established risk factors, including high-density lipoprotein cholesterol, low-density lipoprotein cholesterol, lactate dehydrogenase, alkaline phosphatase and creatine kinase, all of which play crucial roles in the development of heart diseases [16, 17]. Consuming red and processed meat has been linked to several mechanisms that could potentially increase the risk of IHD. For example, a meta-analysis of randomized clinical trials revealed that red meat intake was associated with elevated levels of low-density lipoprotein cholesterol in the bloodstream [18]. Moreover, dietary heme iron found in red meat has also been linked to myocardial infarction and fatal CHD [19]. During meat processing, additional components such as sodium are often added, which have been associated with a significant increase in overall cardiovascular disease risk [20]. In certain animal models, preservatives such as nitrates and nitrate byproducts in processed red meat have been linked to endothelial dysfunction, atherosclerosis and insulin resistance [21]. Although the GBD database lists diets high in red and processed meat as a risk factor for IHD, there is currently no comprehensive literature on the global burden of IHD attributed to high red and processed meat consumption.

Whether consuming red and processed meat is a risk factor for IHD remains debatable. Some early studies found no association between the two [22–25], while others have suggested that the links between processed meat consumption and diabetes or CHD might be due to unhealthy diets or lifestyles rather than any causal effects of processed meats [26]. Additionally, there is a lack of data on cooking methods that could alter the health-related effects of red or processed meats. However, there is also mounting evidence indicating that the consumption of red and processed meat is linked to an elevated risk of cardiometabolic disease [8, 24, 27], which could be due to differences in the definition of high and low intake in each country. For instance, populations in Low-Income Food Deficit Countries with a "high" meat intake (cooked, off-bone, and inedible) may have equivalent intake levels to "low" meat intake population segments in high-meat consumption countries such as America and Europe [28].

This study found that the number of deaths and DALYs attributable to high intake of red meat increased from 1990 to 2019, while ASMR and ASDR decreased. Further analysis showed that the corresponding mortality in some high-middle SDI countries has decreased, which might be related to better medical technology and meat quality testing. However, the death rate in middle and low SDI areas is still increasing. The number of deaths and DALYs due to IHD is increasing globally, particularly in middle and low SDI regions, due to their large population base. It has been reported that the consumption of red meat can also lead to hypertension, obesity, vascular sclerosis and hyperlipidemia, which may further contribute to increased IHD death risk [29].

Our analysis of GBD data revealed that the burden of IHD attributed to diet high in red and processed meat is greater in males than females. The ASMR and ASDR were significantly higher in males than in females, which could be attributed to several factors. First, males consume more red and processed meat than females, with the average intake being 75.9 g/day among males and 55.8 g/day among females [8, 25]. Second, differences in obesity, smoking, hypertension and other risk factors between males and females can lead to higher mortality in males. Third, after menopause, changes in estrogen levels can gradually increase the death rate of IHD in women, even exceeding that of men as they age [30]. Similarly, in our current investigation, we observed a higher incidence of deaths and DALYs in males than females before the age of 75-79 years. This phenomenon may be attributed to the narrowing of sex-based disparities in mortality and DALYs associated with IHD, as women progressively catch up with men in terms of risk of developing heart disease risk.

The impact of red and processed meat on deaths and DALYs due to IHD varied significantly across regions and countries between 1990 and 2019. East Asia had the highest death toll due to a diet high in red meat, with China being the largest contributor. As the Chinese economy has progressed, the dietary habits of Chinese people have undergone significant changes, including higher consumption of protein and fat and lower intake of cereals and vegetables [31]. However, there has been little change in the consumption patterns in high-income countries over the past 50 years. On the other hand, the United States of America had the highest number of deaths due to a diet high in processed meat, which may be related to the American diet including higher amounts of processed meat compared to other countries and a cultural preference for fast and convenient foods that are often high in saturated and trans fats. Additionally, the food industry in the United States of America has been criticized for its aggressive marketing of processed foods and for lobbying against stricter regulations on the production and labeling of processed meats. Meanwhile, according to the American Heart Association's recommendation of approximately 100 grams of meat consumption per week, the average adult in the United States of America exceeds this limit by consuming an average of 105 grams of meat per day [32, 33]. It is estimated that up to 60% of the Western diet consists of high amounts of ultra-processed foods [34], which began in high-income countries but have spread worldwide due to the explosive growth in the manufacture and consumption of ultra-processed foods [35, 36]. Although Eastern Europe had the highest number of deaths due to a diet high in processed meat, the number of deaths, ASMR and ASDR decreased from 1990 to 2019, which might be due to the region's preference for processed meat. At the same time, we noticed that the number of deaths, ASMR and ASDR in several low SDI regions were increasing. Mortality from IHD in Western countries has dramatically decreased throughout the last decades with a greater focus on primary prevention and improved diagnosis and treatment of IHD. In addition, although globalization has been associated with improvements in healthcare systems, the adoption of Western lifestyles has been linked to a higher prevalence of cardiovascular risk factors, thereby representing a significant public health challenge [37, 38].

Our study found positive correlations between the SDI and ASMR of IHD associated with a diet high in red meat when the SDI was less than 0.7. However, as the SDI increased, this correlation decreased substantially. Between 1990 and 2019, the number of deaths attributed to IHD from a diet high in red meat decreased only in regions with high SDI. However, the other four regions with lower SDI showed varying degrees of increase. The significant rise in global consumption of red meat since 1990, which has now reached 184 million tons per year and continues to show an upward trend, primarily in the middle- and low-SDI regions, could explain this observed disparity [22]. As global consumption of red and processed meat increases, the proportion of deaths caused by IHD also increases, indicating that the impact of these meats on IHD may eventually become more severe in the future. A non-linear relationship exists between SDI and the corresponding ASMR of IHD attributed to diet high in processed meat, but only before the SDI reaches 0.6, indicating that processed meat has not yet become a significant food source in regions with low to middle SDI. However, the ASMR of IHD attributed to a diet high in red and processed meat has decreased in regions with high and high-middle SDI, which may reflect better access to health services and improved treatments [39]. Meanwhile, regions with high-middle and high SDI showed the highest number of deaths and DALYs attributed to high red and processed meat consumption in 2019. Therefore, efforts to raise awareness about the adverse effects of red and processed meat on health should continue to strengthen in regions with high and high-middle SDI.

The Sustainable Development Goals 3.4 aims to reduce premature mortality caused by non-communicable diseases by one-third before 2030. To achieve this ambitious goal, it is imperative that every country prioritizes reducing death tolls associated with IHD, which is the leading cause of non-communicable disease-related deaths [40]. While changing people's eating habits can be daunting, governments can help shift their citizens' diets through various means, including education and awareness campaigns, regulation and legislation, subsidies and incentives, public health programs, and partnerships with private organizations. Encouraging healthy eating habits requires a comprehensive and multifaceted approach. It is recommended to enhance the implementation of public health campaigns that focus on primary prevention, supported by a robust primary care infrastructure. These campaigns should be extended to low- and middle-income countries and low socioeconomic status groups in high-income countries [37].

Several limitations should be considered when interpreting the findings of our study. First, the primary data source for all dietary hazards was retrieved from the 24-hour dietary recall surveys, and the data quality might be of concern. Second, while civil registration and statistics systems are critical sources of vital statistics for calculating mortality rates, their population coverage has been disappointing, and this might have affected our estimates. Third, we estimated the risk associated with red and processed meat as a whole, which may not accurately reflect the risk associated with specific types of meat. Fourth, we did not examine whether different types of red and processed meat intake had different effects on the risk of IHD.

## Conclusion

During the period spanning 1990 and 2019, there was an increase in the global number of deaths and DALYs associated with IHD attributed to high intake of red and processed meat, while the ASMR and ASDR exhibited a decreasing trend. Notably, China and the United States of America had the highest number of deaths attributed to high red and processed meat intake. Males accounted for most of the burden of IHD, which increased with age. Although the ASMR of IHD attributed to high intake of red and processed meat decreased in high and high-middle SDI regions, their burden remains high, with regions with below-middle SDI experiencing an increase. These findings have significant implications for formulating targeted interventions and policies for preventing and managing IHD attributed to high red and processed meat intake in various countries and regions.

### Supplementary Information

**Additional file 1. **The deaths number of ischemic heart disease attributed to diet high in red meat in 204 countries.

**Additional file 2. **The ASMR of cardiovascular diseases attributed to diet high in red meat in 204 countries.

**Additional file 3. **The ASDR of cardiovascular diseases attributed to diet high in red meat in 204 countries.

**Additional file 4. **The deaths number of ischemic heart disease attributed to diet high in processed meat in 204 countries.

**Additional file 5. **The ASMR of ischemic heart disease attributed to diet high in processed meat in 204 countries.

**Additional file 6.** The ASDR of ischemic heart disease attributed to diet high in processed meat in 204 countries.

## Data Availability

The datasets presented in this study can be found in online database. The names of the database can be found below: https://vizhub.healthdata.org/gbd-results/.
